# Glycated Hemoglobin, Fasting Insulin and the Metabolic Syndrome in Males. Cross-Sectional Analyses of the Aragon Workers’ Health Study Baseline

**DOI:** 10.1371/journal.pone.0132244

**Published:** 2015-08-04

**Authors:** Gabriela Saravia, Fernando Civeira, Yamilee Hurtado-Roca, Eva Andres, Montserrat Leon, Miguel Pocovi, Jose Ordovas, Eliseo Guallar, Antonio Fernandez-Ortiz, Jose Antonio Casasnovas, Martin Laclaustra

**Affiliations:** 1 Department of Epidemiogy, Atherothrombosis and Imaging, Spanish National Center for Cardiovascular Research (CNIC), Madrid, Spain; 2 Instituto Aragonés de Ciencias de la Salud (I+CS), Zaragoza, Spain; 3 Boca Raton Clinical Research Global Peru, Lima, Peru; 4 Instituto de Investigación 12 de Octubre. CIBER-Epidemiología y Salud Pública, Madrid, Spain; 5 Departments of Epidemiology and Medicine, and Welch Center for Prevention, Epidemiology, and Clinical Research, Johns Hopkins Bloomberg School of Public Health, Baltimore, MD, United States of America; 6 Hospital Clínico San Carlos, Universidad Complutense, Madrid, Spain; 7 Department of Preventive Medicine and Public Health, School of Medicine, Universidad Autonoma de Madrid, Madrid, Spain; 8 Department of Epidemiology, St. Louis University, St Louis, Missouri, United States of America; Virgen Macarena University Hospital, School of Medicine, University of Seville, SPAIN

## Abstract

**Background and Aims:**

Glycated hemoglobin (HbA1c) is currently used to diagnose diabetes mellitus, while insulin has been relegated to research. Both, however, may help understanding the metabolic syndrome and profiling patients. We examined the association of HbA1c and fasting insulin with clustering of metabolic syndrome criteria and insulin resistance as two essential characteristics of the metabolic syndrome.

**Methods:**

We used baseline data from 3200 non-diabetic male participants in the Aragon Workers' Health Study. We conducted analysis to estimate age-adjusted odds ratios (ORs) across tertiles of HbA1c and insulin. Fasting glucose and Homeostatic model assessment - Insulin Resistance were used as reference. Here we report the uppermost-to-lowest tertile ORs (95%CI).

**Results:**

Mean age (SD) was 48.5 (8.8) years and 23% of participants had metabolic syndrome. The ORs for metabolic syndrome criteria tended to be higher across HbA1c than across glucose, except for high blood pressure. Insulin was associated with the criteria more strongly than HbA1c and similarly to Homeostatic model assessment - Insulin Resistance (HOMA-IR). For metabolic syndrome, the OR of HbA1c was 2.68, of insulin, 11.36, of glucose, 7.03, and of HOMA-IR, 14.40. For the clustering of 2 or more non-glycemic criteria, the OR of HbA1c was 2.10, of insulin, 8.94, of glucose, 1.73, and of HOMA-IR, 7.83. All ORs were statistically significant. The areas under the receiver operating characteristics curves for metabolic syndrome were 0.670 (across HbA1c values) and 0.770 (across insulin values), and, for insulin resistance, 0.647 (HbA1c) and 0.995 (insulin). Among non-metabolic syndrome patients, a small insulin elevation identified risk factor clustering.

**Conclusions:**

HbA1c and specially insulin levels were associated with metabolic syndrome criteria, their clustering, and insulin resistance. Insulin could provide early information in subjects prone to develop metabolic syndrome.

## Introduction

The American Diabetes Association included hemoglobin A1c (HbA1c) as a diagnostic test for diabetes in their 2010 clinical practice recommendations [1]. In addition to being tightly associated with diabetes, HbA1c predicts cardiovascular events among non-diabetic individuals and it may outperform fasting plasma glucose for predicting cardiovascular disease [2]. In contrast, serum insulin measurement is not used as often, despite the fact that it can identify insulin-resistant subjects, which is a strong marker of future diabetes [3].

The metabolic syndrome defines a group of cardiovascular risk factors that appear together and are strongly associated with the development of ischemic heart disease and diabetes [4]. The metabolic syndrome is tightly linked to insulin resistance [5], which is considered one of its essential characteristics and it was initially required for diagnosis of the syndrome [6].Still, the clinical criteria that are currently used for diagnosis face numerous limitations [7]. We hypothesize that HbA1c and insulin measurements, readily available in many clinical settings, could be used to improve the profiling of patients with metabolic syndrome or subjects prone to develop it. Previous research has compared HbA1c to glucose and explored the possibility of substituting HbA1c for the glucose criterion or adding HbA1c as an additional diagnostic criterion [8–13] but no study has compared HbA1c and insulin.

In this study we analyze cross-sectional data from the baseline visit of the Aragon Workers’ Health Study (AWHS) [14] and compare HbA1c and insulin in their association with the metabolic syndrome and its traits in non-diabetic individuals. We use risk factor clustering and insulin resistance as comparison metrics and evaluate their potential for identifying higher metabolic risk subgroups.

## Research Design and Methods

### Study population

AWHS (Aragon Workers' Health Study) is a longitudinal cohort study to evaluate cardiovascular risk factors and subclinical atherosclerosis [14] conducted among workers of the General Motors assembly factory in Figueruelas (Zaragoza), Spain. All factory workers attending the annual health exam between February 2009 and May 2010 were invited to participate in the study, of which 5456 consented to participate (94.5% participation rate). Because of the small number of women, we restricted the analysis to males (N = 5104) and because relationships among our variables of interest were different among diabetic patients we further restricted our sample to non-diabetic participants (N = 4806), defining diabetes as fasting glucose ≥7 mmol/L [≥126 mg/dL], HbA1c ≥6.5%, or being on anti-diabetic medication. Among those, 3392 underwent their laboratory measurements when techniques for both parameters, HbA1c and insulin, were available. We excluded those with missing data on variables necessary to diagnose metabolic syndrome (N = 167), or on other relevant covariables such as body mass index (N = 25). The final sample size was 3200. All participants signed a written informed consent and the study was approved by the Ethics Committee for Clinical Investigation of Aragon (CEICA).

### Data collection

Data collection was based on clinical interviews, questionnaires, a physical exam, and a fasting blood draw. Waist circumference was measured at a plane in the midpoint between the ilium and the costal border with the participant standing. We averaged three blood pressure readings measured using an automatic oscillometric sphygmomanometer OMRON M10-IT (OMRON Healthcare Co. Ltd., Kyoto, Japan) with the participant sitting after a 5-min rest.

Peripheral venous blood samples were collected after an 8-hour fasting period. Whole blood HbA1c was measured by reverse-phase cationic exchange chromatography and double wave-length colorimetric quantification (Analyzer ADAMS A1c HA-8160, Arkray Factory). Serum triglycerides, total cholesterol, HDL-cholesterol, and fasting glucose were measured in an ILAB650 analyzer using the manufacturer’s kits based on spectrophotometric assays. Serum insulin was measured in an Access 2 analyzer (Beckman Coulter, CA, USA) using a manufacturer’s ultrasensitive kit based on a double sandwich immunoassay in frozen samples. HOMA-IR (Homeostatic model assessment—Insulin Resistance) was calculated as glucose (in mmol/L) multiplied by insulin (in pmol/L) and divided by 135 [15]. Blood extraction, anthropometric and biochemical measurements were certified with ISO 9001:2008 standards.

### Metabolic syndrome and risk factors clustering

Metabolic syndrome was defined according to the 2009 joint interim statement of several international associations [4], in which subjects must meet at least 3 of the 5 following criteria: high waist circumference (waist circumference ≥102 cm), high triglycerides (≥1.7 mmol/L [≥150 mg/dL] or being on triglycerides lowering medications), low HDL-cholesterol (<1.0 mmol/L [<40 mg/dL]), high blood pressure (systolic blood pressure ≥ 130 mmHg and/or diastolic blood pressure ≥ 85 mmHg or being on antihypertensive medications), and high fasting glucose (≥5.6 mmol/L [≥100 mg/dL] or being on drug treatment for elevated glucose).

HbA1c integrates glucose levels over the previous 3 months and insulin is linked with glucose in a biological feed-back loop. In this project, we compared HbA1c and insulin with fasting glucose and HOMA-IR, which also incorporates glucose levels in its formula. Since we expected that all of these parameters were going to be correlated with glucose levels (one of the variables used in the definition of the metabolic syndrome), in addition to the metabolic syndrome, we also evaluated clusters which do not include fasting glucose: 2-or-more-non-glycemic-criteria and 3-or-more-non-glycemic-criteria defined as the accumulation of, respectively, ≥2 or ≥3 criteria for metabolic syndrome other than high fasting glucose.

### Statistical methods

We evaluated the association of HbA1c and insulin with the presence of the metabolic syndrome components and the metabolic syndrome. We also investigated their association with 2-or-more-non-glycemic-criteria, with 3-or-more-non-glycemic-criteria, and with insulin resistance, defined as HOMA-IR ≥2.6 [16]. The same associations were studied for fasting glucose and HOMA-IR, which are used as comparison reference parameters.

We divided the sample in HbA1c tertiles (cutoff points at 5.3 and 5.5%) and insulin tertiles (cutoff points at 26.4 and 42.6 pmol/L [3.8 and 6.1 uU/mL]). Odds ratios (OR) for the presence and clustering of metabolic syndrome criteria and insulin resistance comparing the higher tertiles of HbA1c and insulin to the lowest tertile were calculated using multivariable logistic regression adjusted for age (continuous). Tests for linear trend were calculated by including HbA1c and insulin as continuous variables in the adjusted models. The same calculations for glycemia and HOMA-IR are provided as reference.

To understand the possible role of body mass index as a confounder on the association of our studied variables with the individual factors and their clustering we created an additional model adjusted for BMI, as well as a variable for clustering of non-glycemic-and-non-anthropometric criteria.

We also calculated the receiver operating characteristic curves (ROC curves) for HbA1c and insulin to evaluate their discrimination ability for detecting metabolic syndrome components, metabolic syndrome, clusters of non-glycemic components of the metabolic syndrome and insulin resistance, alongside with those for glycemia and HOMA-IR for comparison.

Using the same HbA1c and insulin cutoffs, we stratified the analysis by presence of metabolic syndrome. As a sensitivity analysis we verified that the estimates did not substantially change when adjusting them for 5-year-wide age categories instead of continuous-age.

Statistical analyses were performed with R (version 3.0.2) [17] and the plots were created using the ROCR package (version 1.0–5).

## Results

The mean (SD) age of the 3200 non-diabetic men was 48.5 (8.8) years (Table 1). Metabolic syndrome was diagnosed in 23.0% of participants and 11.8% were insulin resistant, but only 7.3% of participants had both metabolic syndrome and insulin resistance. The metabolic syndrome had 62.0% sensitivity and 82.2% specificity for detecting insulin resistance.

**Table 1:** Characteristics of the study population with and without metabolic syndrome.

	Overall	Without metabolic syndrome	With metabolic syndrome	p
**N**	3200	2465	735	
**Age (years)**	48.5(8.8)	47.5(9.4)	51.8(5.2)	<0.001
**Body mass index (Kg/m** ^**2**^ **)**	27.5(3.5)	26.6(3.0)	30.6(3.5)	<0.001
**Waist circumference (cm)**	96.7(9.7)	94.1(8.5)	105.6(8.4)	<0.001
**Total cholesterol (mmol/L)**	5.49(0.98)	5.43(0.98)	5.70(0.96)	<0.001
**Triglycerides (mmol/L)**	1.58(1.02)	1.37(0.82)	2.31(1.26)	<0.001
**HDL-cholesterol (mmol/L)**	1.37(0.28)	1.41(0.28)	1.23(0.26)	<0.001
**LDL-cholesterol (mmol/L)** *	3.41(0.83)	3.39(0.83)	3.46(0.80)	0.08
**Systolic BP** † **(mmHg)**	126.3(14.3)	124.0(13.2)	134.3(15.0)	<0.001
**Diastolic BP** † **(mmHg)**	83.3(9.8)	81.4(9.3)	89.8(8.8)	<0.001
**Fasting glucose (mmol/L)**	5.30(0.61)	5.17(0.56)	5.71(0.59)	<0.001
**HOMA-IR** ‡	1.6(1.2)	1.3(0.9)	2.5(1.6)	<0.001
**Hemoglobin A1c (%)**	5.4(0.3)	5.4(0.3)	5.5(0.3)	<0.001
**Insulin (pmol/L)**	39.9(26.5)	34.5(20.9)	58.0(34.2)	<0.001
**Clinical hypertension (%)**	27.6	19.9	53.3	<0.001

Average and standard deviation of clinical, physical, and biochemical parameters. P values were calculated from t-tests.

*n = 3136 due to missing values.

^†^BP: Blood Pressure.

^‡^HOMA-IR: Homeostatic Model Assessment—Insulin Resistance.

**Fig 1:**
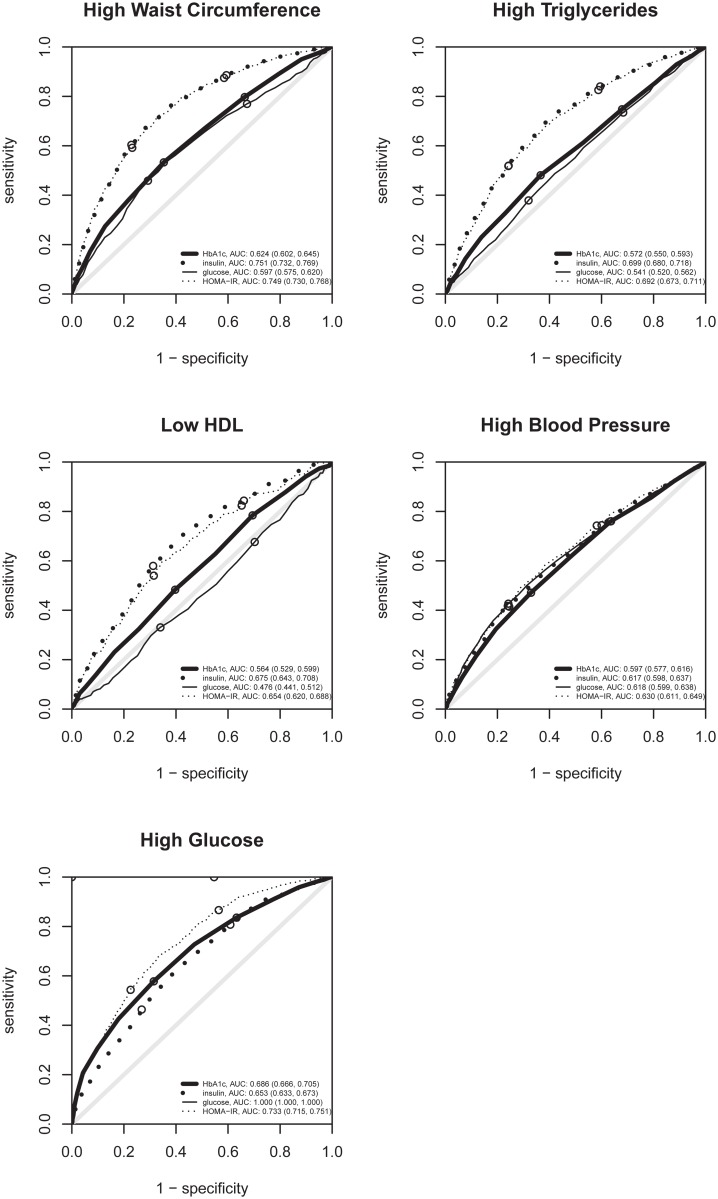
ROC curves of HbA1c, insulin, glycemia and HOMA-IR for metabolic syndrome criteria. ROC curves for detecting individually each metabolic syndrome criteria. The small circles indicate the sensitivity and specificity when using the tertiles of each variable as cut-offs. For high glucose, the glycemia curve can not be seen as it lies exactly at the left and upper borders.

**Fig 2:**
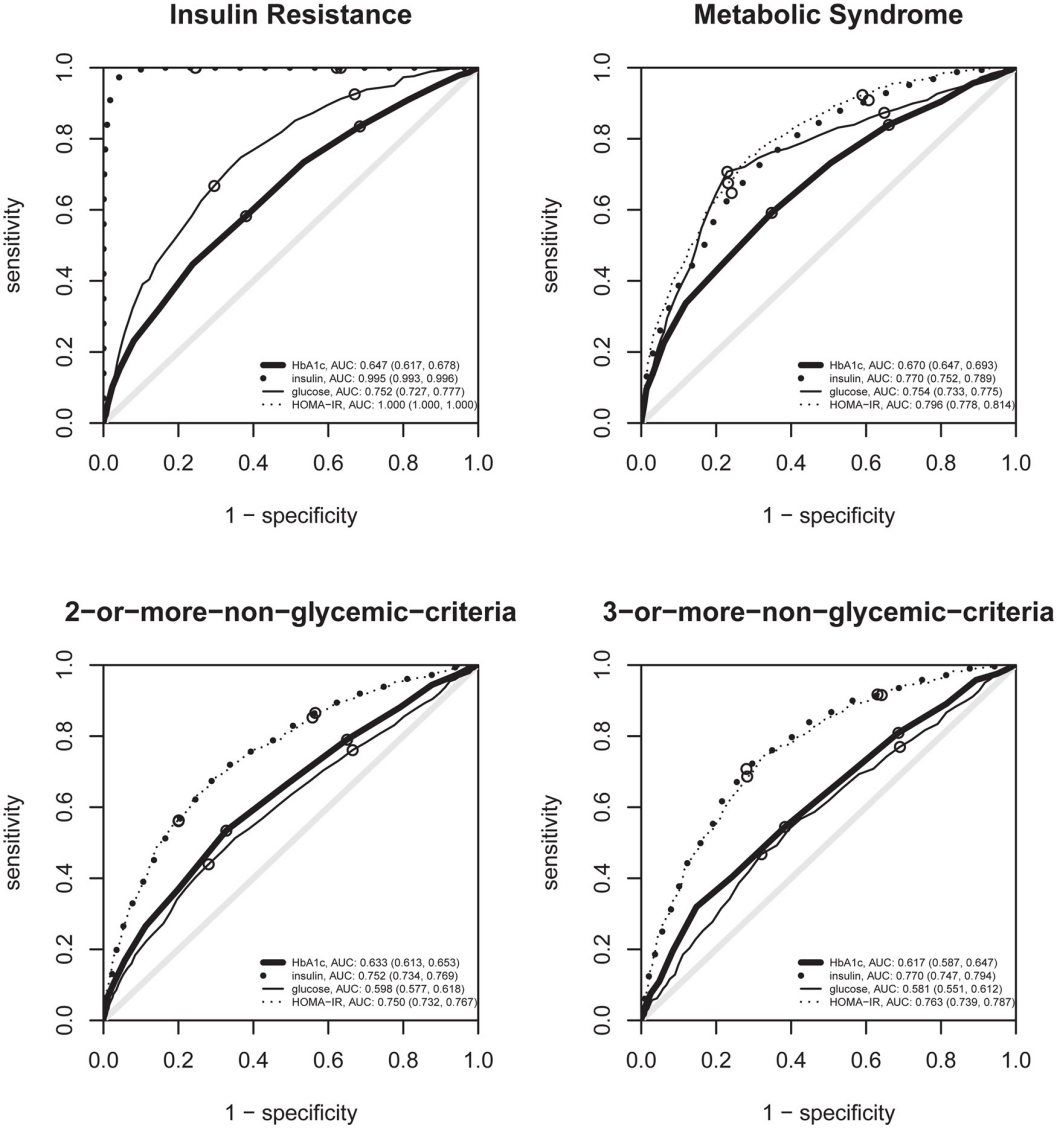
ROC curves of HbA1c, insulin, glycemia and HOMA-IR for insulin resistance and criteria clusters. ROC curves for detecting insulin resistance, the metabolic syndrome, 2 or more, and 3 or more criteria for the metabolic syndrome other than the high glucose criterion. The small circles indicate the sensitivity and specificity when using the tertiles of each variable as cut-offs. For insulin resistance, the HOMA-IR curve can not be seen as it lies exactly at the left and upper borders.

The odds of having metabolic syndrome increased by a factor of 7.16 (95%CI: 5.14, 10.01) per unit of HbA1c. Glycated hemoglobin (HbA1c) also was associated with HOMA-IR: per each HbA1c unit increase, HOMA-IR was multiplied by 1.72 (95%CI: 1.60, 1.85). Compared to participants in the lower tertile of HbA1c, those in the upper tertile had a higher prevalence of all diagnostic criteria for the metabolic syndrome, their clustering in the metabolic syndrome, the clustering of factors other than high glucose (2-or-more-non-glycemic-criteria and 3-or-more-non-glycemic-criteria), and insulin resistance (Table 2). Odds ratios for diagnostic criteria for the metabolic syndrome for HbA1c tended to be higher than those for fasting glucose, except for high blood pressure. The odds ratios for HbA1c were also higher than those of fasting glucose for non-glycemic-criteria clusters. HbA1c tended to perform better than glucose in discriminating these conditions, as seen in ROC curves (Figs 1 and 2).

**Table 2:** Association of HbA1c, insulin, glucose, and HOMA-IR tertiles with metabolic syndrome criteria.

		HbA1c			Insulin			Glucose			HOMA-IR*	
		tertiles			tertiles			tertiles			tertiles	
		(%)			(pmol/L)			(mmol/L)				
	< 5.3	≥ 5.3 and < 5.5	≥ 5.5	< 26.4	≥ 26.4 and < 42.6	≥ 42.6	< 5.00	≥ 5.00 and < 5.55	≥ 5.55	< 1.029	≥ 1.029 and < 1.688	≥ 1.688
**N**	954	953	1293	1036	1093	1071	959	1155	1086	1067	1066	1067
**Average**	5.08	5.35	5.66	19.33	33.71	66.17	4.61	5.24	5.96	0.73	1.33	2.75
**High Waist Circumf. (%)**	**19.2**	**25.1**	**37.3**	**9.9**	**23.3**	**51.0**	**21.7**	**24.3**	**38.2**	**10.6**	**24.0**	**50.1**
OR †	1.00	1.22	1.98	1.00	2.67	9.13	1.00	1.08	1.82	1.00	2.56	7.90
	(Reference)	(0.97,1.52)	(1.62,2.43)	(Reference)	(2.08,3.43)	(7.22,11.63)	(Reference)	(0.88,1.33)	(1.49,2.23)	(Reference)	(2.01,3.27)	(6.29,9.99)
**High Triglycerid. (%)**	**28.0**	**29.7**	**39.4**	**16.3**	**31.1**	**51.4**	**29.3**	**32.6**	**37.0**	**17.3**	**30.6**	**51.5**
OR	1.00	0.99	1.44	1.00	2.26	5.25	1.00	1.11	1.23	1.00	2.05	4.80
	(Reference)	(0.81,1.21)	(1.19,1.74)	(Reference)	(1.84,2.79)	(4.28,6.45)	(Reference)	(0.92,1.34)	(1.01,1.49)	(Reference)	(1.67,2.52)	(3.93,5.87)
**Low HDL-cholest. (%)**	**6.1**	**8.5**	**10.1**	**4.1**	**6.5**	**14.6**	**9.1**	**8.1**	**8.2**	**4.5**	**7.1**	**13.6**
OR	1.00	1.55	1.97	1.00	1.68	4.18	1.00	0.89	0.93	1.00	1.67	3.53
	(Reference)	(1.09,2.23)	(1.40,2.79)	(Reference)	(1.14,2.50)	(2.96,6.02)	(Reference)	(0.65,1.21)	(0.68,1.29)	(Reference)	(1.16,2.45)	(2.52,5.01)
**High Blood Pressure (%)**	**42.3**	**51.2**	**61.5**	**41.6**	**50.9**	**65.4**	**42.2**	**48.7**	**66.3**	**40.7**	**51.5**	**66.0**
OR	1.00	1.10	1.43	1.00	1.37	2.48	1.00	1.16	1.94	1.00	1.45	2.49
	(Reference)	(0.91,1.34)	(1.19,1.72)	(Reference)	(1.15,1.64)	(2.06,2.98)	(Reference)	(0.97,1.39)	(1.61,2.35)	(Reference)	(1.21,1.74)	(2.07,2.99)
**High Glucose (%)**	**18.7**	**29.4**	**48.6**	**20.2**	**34.1**	**47.1**	**–**	**–**	**–**	**13.6**	**32.9**	**55.3**
OR	1.00	1.50	3.07	1.00	1.98	3.34	–	–	–	1.00	3.02‡	7.35‡
	(Reference)	(1.21,1.88)	(2.51,3.77)	(Reference)	(1.62,2.43)	(2.74,4.08)				(Reference)	(2.42,3.77)	(5.93,9.16)
**Metabolic Syndrome (%)**	**12.4**	**19.1**	**33.6**	**6.5**	**17.6**	**44.4**	**9.7**	**10.6**	**47.9**	**5.3**	**17.1**	**46.5**
OR	1.00	1.38	2.68	1.00	2.97	11.36	1.00	1.02‡	7.03‡	1.00	3.47‡	14.40‡
	(Reference)	(1.07,1.79)	(2.13,3.39)	(Reference)	(2.22,4.01)	(8.65,15.13)	(Reference)	(0.77,1.36)	(5.50,9.06)	(Reference)	(2.55,4.78)	(10.80,19.53)
**2+-non-glyc.-criteria (%)**	**26.0**	**31.8**	**48.9**	**15.3**	**32.8**	**62.2**	**29.5**	**32.9**	**47.9**	**16.4**	**32.4**	**62.1**
OR	1.00	1.12	2.10	1.00	2.61	8.94	1.00	1.08	1.73	1.00	2.34	7.83
	(Reference)	(0.91,1.38)	(1.74,2.54)	(Reference)	(2.11,3.24)	(7.24,11.09)	(Reference)	(0.89,1.30)	(1.43,2.10)	(Reference)	(1.90,2.89)	(6.38,9.65)
**3+-non-glyc.-criteria (%)**	**8.1**	**11.2**	**17.0**	**3.3**	**7.7**	**26.7**	**9.7**	**10.6**	**17.4**	**3.2**	**8.7**	**26.0**
OR	1.00	1.25	1.87	1.00	2.36	10.20	1.00	1.03	1.62	1.00	2.78	9.84
	(Reference)	(0.92,1.72)	(1.42,2.50)	(Reference)	(1.58,3.59)	(7.16,14.99)	(Reference)	(0.77,1.37)	(1.24,2.13)	(Reference)	(1.87,4.21)	(6.90,14.48)
**Insulin Resistance (%)**	**6.5**	**10.0**	**16.9**	**0.0**	**0.0**	**35.1**	**2.9**	**8.4**	**23.1**	**–**	**–**	**–**
OR	1.00	1.50	2.66	–	–	–‡	1.00	3.03‡	9.84‡	–	–	–
	(Reference)	(1.07,2.11)	(1.97,3.64)				(Reference)	(2.00,4.74)	(6.64,15.13)			

Adjusted odds ratios and their confidence interval calculated from a logistic regression model adjusted for age (continuous). All tests for linear trend, calculated from models introducing each predictor as continuous variable, were statistically significant at a level α<0.001 except for glucose and high triglycerides (p = 0.03) and for glucose and Low HDL-cholesterol (p = 0.31).

*HOMA-IR: Homeostatic Model Assessment—Insulin Resistance.

^†^OR: Odds Ratio.

^‡^Outcome variable directly related to the predictor because of a formula or because of a diagnostic criterion.

Per each 10 pmol/L (1.4 uU/mL) increase in insulin, the odds for metabolic syndrome increased by 1.43 (95%CI: 1.38, 1.49). Compared to participants in the lower tertile of insulin, those in the upper tertile had a higher prevalence of all criteria and clusters, with particularly strong intensity for high waist circumference (OR 9.13) and for the clusters (Table 2). These associations were all higher than those for fasting glucose or HbA1c, and tended to be higher than those for HOMA-IR, except for high blood pressure, which was very similar, and for high glucose and metabolic syndrome, which tended to be lower. Insulin tended to perform better than HOMA-IR in discriminating all the criteria of the metabolic syndrome except for high blood pressure and high glucose, but the latter is linked with HOMA-IR by formula (Fig 1). The areas under the ROC curve of HbA1c, insulin, glycemia, and HOMA-IR for metabolic syndrome were 0.670, 0.770, 0.754, and 0.796, respectively (Fig 2). For non-glycemic-criteria clusters, the areas under the ROC curve were greater or very similar for insulin than for HOMA-IR. For the insulin resistance threshold used, insulin was far more relevant than glucose in discriminating insulin resistance, carrying almost all the necessary information (area under curve 0.995) to discriminate insulin resistance with high sensitivity and specificity (Fig 2).

Among participants with metabolic syndrome (N = 735), high HbA1c was mainly associated with high glucose and insulin resistance while high insulin was additionally associated with high waist circumference (Table A in S1 File). In the absence of metabolic syndrome, insulin was more strongly associated than HbA1c to all criteria except high glucose and there were significant differences between the low- and mid-range of insulin for all criteria except high blood pressure (Table B in S1 File).

The rank order of the strengths of association of HbA1c, insulin, glucose, and HOMA-IR with the criteria and clusters remained the same after adjusting the models for body mass index (Table C in S1 File).

## Discussion

In this analysis of non-diabetic male participants from the AWHS study we found that HbA1c was associated with non-glycemic metabolic syndrome components and their clustering more closely than glucose. Moreover, insulin outperformed glucose and HbA1c in its association with the metabolic syndrome, its criteria and clusters of non-glycemic criteria. Prevalence of various metabolic syndrome criteria increased among participants without metabolic syndrome who had even only mild insulin elevation. Finally, insulin alone had a discriminating ability similar to that of HOMA-IR, indicating that glucose contributes little information to the HOMA-IR formula in contexts similar to our population.

HbA1c is a product of non-enzymatic glycosylation that reflects the average glucose over the preceding weeks [18]. HbA1c measurement does not require fasting and is highly standardized and widely available nowadays [1]. Insulin measurement has a similar cost, but its use has been confined to the clinical diagnosis of selected endocrine conditions, like insulinomas [19], and to the measurement of insulin resistance [20], mostly for research purposes. Current clinical guidelines discourage measuring insulin concentration in the assessment of cardiometabolic risk, because knowledge of this value does not alter patient management in a prevention scheme that focuses in treatment of high-risk patients [19], in spite of the strong known independent association of insulin with ischemic heart disease [21]. Insulin assays are still in a process of standardization [22] and fasting blood samples are needed to calculate HOMA-IR [23].

HbA1c was associated with high waist circumference, high triglycerides, and low HDL-cholesterol more closely than glucose in our results. Succurro et al. had also reported a better correlation of HbA1c with measures of visceral obesity, HDL-cholesterol, and triglycerides [8]. Like in our study, in Sucurro et al.’s analyses, glucose correlated better than HbA1c with systolic blood pressure and pulse pressure, suggesting that different pathophysiological pathways underlie the clustering of blood pressure with other metabolic parameters. Indeed, the complex pathophysiology of the metabolic syndrome conveys separate vasomotor and lipid pathways [5]. Hence, it is important to know which aspects of the syndrome are captured by glucose, which by HbA1c, and which by insulin. HbA1c is a better predictor than glucose for atherosclerotic events [2], which are strongly linked to lipid metabolism [24]. Furthermore, flow-mediated dilation, a measurement of endothelial dysfunction recognized as an early stage of atherosclerosis, is more strongly associated with HbA1c than with glucose [25]. We found that insulin was strongly associated to all traits individually more intensely than HbA1c and glucose and, in particular, to abdominal obesity. The metabolic syndrome was linked to insulin resistance since the concept was introduced. Interestingly, our results show that insulin alone was similarly or more strongly associated than HOMA-IR to all the traits excluding high glucose, which is included in the HOMA-IR formula.

Using HbA1c to identify metabolic syndrome patients yielded 0.670 as the area under ROC curve. Our result is similar to 0.678, previously reported by Succurro et al. [8], and 0.648, previously reported by Sung et al. [9], and greater than 0.602, reported by Dilley et al. [26]. The area under curve that we found for insulin, 0.770, was greater than that for HbA1c.

Some researchers have tried to substitute HbA1c for the glucose metabolic syndrome criterion or to add HbA1c as an additional diagnostic criterion [8,10–13]. Succurro et al.[8] pointed out that, in their analysis, the metabolic syndrome using an HbA1c criterion instead of glucose performed worse in detecting some subjects who still had an unfavorable cardiometabolic risk profile. Using the current definition of the metabolic syndrome (which includes glucose) as standard to compare the glucose criterion with criteria based on other measurements is inconclusive. In the absence of a clinical gold-standard, measurements that are closer to the syndrome pathophysiology should be used to evaluate diagnostic performance improvements: Clustering may reflect an underlying common mechanism for the different traits and insulin resistance has been often used as gold-standard for the metabolic syndrome. HbA1c and especially insulin were more strongly associated to clusters of non-glycemic metabolic traits than glucose. Insulin seems to be a good marker for a common underlying mechanism, very similar to HOMA-IR, and HbA1c still seems to be a better marker than glucose. Insulin alone showed a high accuracy for defining insulin resistance in this range of values whereas glucose played a minor role in the HOMA-IR variation. This fact has been known for long [27] but it is very often overlooked. Consequently, if a laboratory measurement was to be considered to be added to the metabolic syndrome criteria, our findings and other pathophysiologic arguments would favor adding insulin over HbA1c, as it was proposed in the initial WHO definitions of the metabolic syndrome [6].

In this otherwise-healthy population, with some overweight but not severe obesity, insulin seems to capture an earlier stage of cardiometabolic disorders than HbA1c and glucose. Insulin appears to be an early marker of metabolic derailment which could identify stages at which correction of lifestyle might require subtler and thus easier modifications than in complete metabolic syndrome patients. This conditions may be missed if only glucose is monitored, as elevation in the fasting period only occur when many other disorders, mainly lipid changes, but probably also hemodynamic, have already taken place. Although insulin measurement does not add discriminative capacity to the metabolic syndrome as a predictor for future diabetes in the general population [28], our results suggest that it might be better than HbA1c and glucose in detecting the initial clustering of cardiovascular risk factors even with small insulin elevations. HbA1c, being a glucose-related measurement does not help shortening the time to detection.

In a non-selected population, an HbA1c in the higher normal range can trigger a search for other cardiometabolic risk factors and induce screening for metabolic syndrome criteria. With a similar cost, however, fasting insulin provides a measurement that may be more closely linked to the pathways conducing to the metabolic syndrome. Given that type 2 diabetes is part of a continuum in which cardiovascular events are among the most severe complications, insulin or insulin resistance may be more valuable measurements in asymptomatic patients than HbA1c for assessing the degree of metabolic impairment [29]. Furthermore, fasting serum insulin can be used as a surrogate of insulin resistance in epidemiological studies [3] particularly among non-diabetic subjects [30].

Nowadays, insulin measurements are used mainly for research purposes [22], insulin is not included in the current definitions of metabolic syndrome or diabetes, despite the fact that many studies documented the association of increased levels of insulin to clustering of cardiovascular risk factors and it was used to define insulin resistance in the initial definitions of the metabolic syndrome[31–35]. Our findings show that insulin could be used as biomarker for cardiovascular risk factors development yielding more information than glucose levels. Consequently, new clinical applications for insulin could be considered and tested. Still, in spite of the promising advantages of insulin that we found, it is early to recommend its use in the clinical setting. Clinical studies should be performed, testing its usefulness for early diagnosis of cardiovascular and metabolic risk and subsequent preventive care. These studies should focus on its role in the prevention of the development of diabetes, by detecting pre-diabetic conditions and hard cardiovascular outcomes, by detecting early risk factors clustering. This would pave the way for insulin to have clinical utility as an early biomarker in primary preventive care settings.

The strengths of this study include the size of a cohort of well characterized participants using high quality procedures including blood extraction, anthropometric and biochemical measurements certified with ISO 9001:2008 standards. Among its limitations, the sample was restricted to working white male participants and the results might not apply to other populations. Additionally, they only apply to non-diabetic people. We excluded participants with diabetes because among those receiving antidiabetic medications, both main parameters considered, HbA1c and insulin, would be altered depending on their treatment and also because, among untreated diabetic patients, the linearity of the relationship between HbA1c and other parameters disappears, which would have led to biased estimates had we included them. The availability of HbA1c and insulin measurements in the laboratory started and ended at different dates for each parameter, which caused that in one third of our participants they did not overlap. Given that the dates and order of the participants attending their annual checkup can be considered random and unrelated to their health status the resulting subsample cannot be considered to be biased or to have a reduced representativeness. We also used a cross-sectional design and thus no temporal criterion of causation can be inferred for these associations. Finally, we used a single measurement of cardiometabolic parameters subject to within personal variability and laboratory measurement error, which may attenuate the observed associations.

## Conclusions

In summary, HbA1c and insulin measurements were highly associated with metabolic syndrome traits. HbA1c is more readily available than insulin and outperforms glucose in terms of association with risk factors, clusters and cardiovascular risk. Fasting insulin is an important measurement for evaluating metabolic risk in research studies and it may be even more informative than HbA1c. Insulin could provide early information in subjects prone to develop metabolic syndrome.

## Supporting Information

S1 File Table A. Association of HbA1c and insulin groups with metabolic traits among participants with metabolic syndrome. Table B. Association of HbA1c and insulin groups with metabolic traits among participants without metabolic syndrome. Table C. Association of HbA1c, insulin, glucose, and HOMA-IR tertiles with metabolic syndrome criteria.
(DOCX)
