# Supplementary material for: Glycated Hemoglobin, Fasting Insulin and the Metabolic Syndrome in Males. Cross-Sectional Analyses of the Aragon Workers’ Health Study Baseline
Source: PLoS One. 2015 Aug 4;10(8):e0132244. doi: 10.1371/journal.pone.0132244 (PMC4524641; doi:10.1371/journal.pone.0132244)
Supplement: S1 File — Table A. Association of HbA1c and insulin groups with metabolic traits among participants with metabolic syndrome. Table B. Association of HbA1c and insulin groups with metabolic traits among participants without metabolic syndrome. Table C. Association of HbA1c, insulin, glucose, and HOMA-IR tertiles with metabolic syndrome criteria. (DOCX) [file pone.0132244.s001.docx]

**S1 Table A.** Association of HbA1c and insulin groups with metabolic traits among participants with metabolic syndrome

|  |  |  | **HbA1c groups** |  |  |  |  | **Insulin groups** |  |  |
| --- | --- | --- | --- | --- | --- | --- | --- | --- | --- | --- |
|  |  | **< 5.3 %** | **≥ 5.3 and < 5.5 %** | **≥ 5.5 %** | **p** |  | **< 26.4 pmol/L** | **≥ 26.4 and < 42.6 pmol/L** | **≥ 42.6 pmol/L** | **p** |
| **N** |  | 118 | 182 | 435 |  |  | 67 | 192 | 476 |  |
| **Average** |  | 5.09 | 5.36 | 5.72 |  |  | 21.60 | 34.88 | 72.44 |  |
| **High Waist Circumference (%)** |  | **74.6** | **72.0** | **74.7** |  |  | **58.2** | **66.1** | **79.4** |  |
| OR* |  | 1.00 | 0.87 | 0.98 | 0.54 |  | 1.00 | 1.44 | 2.83 | <0.001 |
|  |  | (Reference) | (0.51,1.46) | (0.61,1.56) |  |  | (Reference) | (0.81,2.54) | (1.64,4.81) |  |
| **High Triglycerides (%)** |  | **79.7** | **75.3** | **70.8** |  |  | **79.1** | **68.8** | **74.4** |  |
| OR |  | 1.00 | 0.79 | 0.65 | 0.07 |  | 1.00 | 0.55 | 0.74 | 0.61 |
|  |  | (Reference) | (0.44,1.38) | (0.39,1.05) |  |  | (Reference) | (0.28,1.05) | (0.38,1.35) |  |
| **Low HDL-cholesterol (%)** |  | **21.2** | **30.2** | **21.8** |  |  | **28.4** | **18.2** | **25.4** |  |
| OR |  | 1.00 | 1.72 | 1.18 | 0.56 |  | 1.00 | 0.51 | 0.80 | 0.17 |
|  |  | (Reference) | (1.00,3.05) | (0.71,1.99) |  |  | (Reference) | (0.27,0.99) | (0.45,1.44) |  |
| **High Blood Pressure (%)** |  | **93.2** | **86.3** | **90.3** |  |  | **89.6** | **94.8** | **87.8** |  |
| OR |  | 1.00 | 0.45 | 0.65 | 0.50 |  | 1.00 | 2.21 | 0.87 | 0.52 |
|  |  | (Reference) | (0.18,0.99) | (0.27,1.35) |  |  | (Reference) | (0.77,6.02) | (0.35,1.88) |  |
| **High Glucose (%)** |  | **55.1** | **61.0** | **79.1** |  |  | **65.7** | **70.8** | **71.4** |  |
| OR |  | 1.00 | 1.26 | 2.98 | <0.001 |  | 1.00 | 1.33 | 1.35 | 0.04 |
|  |  | (Reference) | (0.78,2.02) | (1.93,4.60) |  |  | (Reference) | (0.72,2.39) | (0.78,2.31) |  |
| **Insulin Resistance (%)** |  | **16.9** | **28.0** | **37.2** |  |  | **0.0** | **0.0** | **48.9** |  |
| OR |  | 1.00 | 1.95 | 3.07 | <0.001 |  | 1.00 | 0.97 | > 10^6^ | <0.001 |
|  |  | (Reference) | (1.11,3.56) | (1.85,5.31) |  |  | (Reference) | - | - |  |

Adjusted odds ratios and confidence intervals calculated from logistic regression models adjusted for age (continuous). P trend values are calculated from a model introducing each predictor as continuous variable. Insulin resistance was defined as HOMA-IR ≥ 2.6. *OR: Odds Ratio.

**S1 Table B.** Association of HbA1c and insulin groups with metabolic traits among participants without metabolic syndrome

|  |  |  | **HbA1c groups** |  |  |  |  | **Insulin groups** |  |  |
| --- | --- | --- | --- | --- | --- | --- | --- | --- | --- | --- |
|  |  | **< 5.3 %** | **≥ 5.3 and < 5.5 %** | **≥ 5.5 %** | **p** |  | **< 26.4 pmol/L** | **≥ 26.4 and < 42.6 pmol/L** | **≥ 42.6 pmol/L** | **p** |
| **N** |  | 836 | 771 | 858 |  |  | 969 | 901 | 595 |  |
| **Average** |  | 5.08 | 5.35 | 5.63 |  |  | 19.17 | 33.46 | 61.15 |  |
| **High Waist Circumference (%)** |  | **11.4** | **14.0** | **18.3** |  |  | **6.6** | **14.2** | **28.2** |  |
| OR* |  | 1.00 | 1.16 | 1.51 | 0.001 |  | 1.00 | 2.29 | 5.55 | <0.001 |
|  |  | (Reference) | (0.86,1.57) | (1.14,2.02) |  |  | (Reference) | (1.68,3.16) | (4.09,7.62) |  |
| **High Triglycerides (%)** |  | **20.7** | **18.9** | **23.5** |  |  | **12.0** | **23.1** | **33.1** |  |
| OR |  | 1.00 | 0.85 | 1.09 | 0.51 |  | 1.00 | 2.18 | 3.62 | <0.001 |
|  |  | (Reference) | (0.66,1.09) | (0.86,1.39) |  |  | (Reference) | (1.71,2.81) | (2.80,4.70) |  |
| **Low HDL-cholesterol (%)** |  | **3.9** | **3.4** | **4.1** |  |  | **2.4** | **4.0** | **5.9** |  |
| OR |  | 1.00 | 1.05 | 1.50 | 0.07 |  | 1.00 | 1.80 | 2.67 | 0.005 |
|  |  | (Reference) | (0.61,1.80) | (0.88,2.55) |  |  | (Reference) | (1.06,3.11) | (1.57,4.64) |  |
| **High Blood Pressure (%)** |  | **35.2** | **42.9** | **46.9** |  |  | **38.3** | **41.5** | **47.4** |  |
| OR |  | 1.00 | 1.08 | 1.08 | 0.85 |  | 1.00 | 1.08 | 1.44 | 0.001 |
|  |  | (Reference) | (0.87,1.34) | (0.88,1.34) |  |  | (Reference) | (0.89,1.31) | (1.16,1.78) |  |
| **High Glucose (%)** |  | **13.5** | **21.9** | **33.1** |  |  | **17.0** | **26.3** | **27.6** |  |
| OR |  | 1.00 | 1.49 | 2.35 | <0.001 |  | 1.00 | 1.68 | 1.85 | <0.001 |
|  |  | (Reference) | (1.14,1.95) | (1.83,3.04) |  |  | (Reference) | (1.34,2.12) | (1.44,2.38) |  |
| **2+-non-glyc.-criteria (%)** |  | **15.6** | **15.7** | **23.0** |  |  | **9.5** | **18.4** | **31.9** |  |
| OR |  | 1.00 | 0.91 | 1.37 | 0.01 |  | 1.00 | 2.10 | 4.47 | <0.001 |
|  |  | (Reference) | (0.69,1.20) | (1.06,1.77) |  |  | (Reference) | (1.60,2.78) | (3.40,5.91) |  |
| **Insulin Resistance (%)** |  | **5.0** | **5.7** | **6.6** |  |  | **0.0** | **0.0** | **24.0** |  |
| OR |  | 1.00 | 1.10 | 1.26 | 0.09 |  | 1.00 | 0.99 | > 10^6^ | <0.001 |
|  |  | (Reference) | (0.71,1.71) | (0.82,1.95) |  |  | (Reference) | - | - |  |

Adjusted odds ratios and confidence intervals calculated from logistic regression models adjusted for age (continuous). P trend values are calculated from a model introducing each predictor as continuous variable. Insulin resistance was defined as HOMA-IR ≥ 2.6. *OR: Odds Ratio.

**S1 Table C.** Association of HbA1c, insulin, glucose, and HOMA-IR tertiles with metabolic syndrome criteria

|  |  |  | **HbA1c tertiles**  **(%)** |  |  |  | **Insulin tertiles**  **(pmol/L)** |  |  |  | **Glucose tertiles**  **(mmol/L)** |  |  |  | **HOMA-IR* tertiles** |  |
| --- | --- | --- | --- | --- | --- | --- | --- | --- | --- | --- | --- | --- | --- | --- | --- | --- |
|  |  | **< 5.3** | **≥ 5.3 and  < 5.5** | **≥ 5.5** |  | **< 26.4** | **≥ 26.4 and  < 42.6** | **≥ 42.6** |  | **< 5.00** | **≥ 5.00 and  < 5.55** | **≥ 5.55** |  | **< 1.029** | **≥ 1.029 and  < 1.688** | **≥ 1.688** |
| **N** |  | 954 | 953 | 1293 |  | 1036 | 1093 | 1071 |  | 959 | 1155 | 1086 |  | 1067 | 1066 | 1067 |
| **Average** |  | 5.08 | 5.35 | 5.66 |  | 19.33 | 33.71 | 66.17 |  | 4.61 | 5.24 | 5.96 |  | 0.73 | 1.33 | 2.75 |
| **High Waist Circumf. (%)** |  | 19.2 | 25.1 | 37.3 |  | **9.9** | **23.3** | **51.0** |  | **21.7** | **24.3** | **38.2** |  | **10.6** | **24.0** | **50.1** |
| OR † |  | 1 | 1.21 | 1.27 |  | 1 | 1.4 | 2.1 |  | 1 | 0.86 | 1.06 |  | 1 | 1.37 | 1.89 |
|  |  | (Reference) | (0.88,1.67) | (0.94,1.72) |  | (Reference) | (1.01,1.95) | (1.53,2.91) |  | (Reference) | (0.63,1.16) | (0.78,1.43) |  | (Reference) | (0.99,1.90) | (1.38,2.59) |
| **High Triglycerid. (%)** |  | 28 | 29.7 | 39.4 |  | 16.3 | 31.1 | 51.4 |  | 29.3 | 32.6 | 37 |  | 17.3 | 30.6 | 51.5 |
| OR |  | 1 | 0.95 | 1.23 |  | 1 | 2.05 | 4.16 |  | 1 | 1.05 | 1.02 |  | 1 | 1.84 | 3.76 |
|  |  | (Reference) | (0.77,1.17) | (1.02,1.50) |  | (Reference) | (1.66,2.54) | (3.34,5.21) |  | (Reference) | (0.87,1.28) | (0.84,1.25) |  | (Reference) | (1.49,2.27) | (3.04,4.68) |
| **Low HDL-cholest. (%)** |  | 6.1 | 8.5 | 10.1 |  | 4.1 | 6.5 | 14.6 |  | 9.1 | 8.1 | 8.2 |  | 4.5 | 7.1 | 13.6 |
| OR |  | 1 | 1.51 | 1.69 |  | 1 | 1.53 | 3.33 |  | 1 | 0.84 | 0.77 |  | 1 | 1.5 | 2.7 |
|  |  | (Reference) | (1.06,2.17) | (1.20,2.41) |  | (Reference) | (1.03,2.29) | (2.29,4.93) |  | (Reference) | (0.61,1.14) | (0.55,1.06) |  | (Reference) | (1.03,2.19) | (1.88,3.95) |
| **High Blood Pressure (%)** |  | 42.3 | 51.2 | 61.5 |  | 41.6 | 50.9 | 65.4 |  | 42.2 | 48.7 | 66.3 |  | 40.7 | 51.5 | 66 |
| OR |  | 1 | 1.06 | 1.22 |  | 1 | 1.14 | 1.62 |  | 1 | 1.1 | 1.64 |  | 1 | 1.21 | 1.65 |
|  |  | (Reference) | (0.87,1.30) | (1.01,1.47) |  | (Reference) | (0.95,1.37) | (1.32,1.99) |  | (Reference) | (0.91,1.33) | (1.35,2.00) |  | (Reference) | (1.00,1.46) | (1.35,2.02) |
| **High Glucose (%)** |  | 18.7 | 29.4 | 48.6 |  | 20.2 | 34.1 | 47.1 |  | **–** | **–** | **–** |  | 13.6 | 32.9 | 55.3 |
| OR |  | 1 | 1.48 | 2.8 |  | 1 | 1.82 | 2.74 |  | – | – | – |  | 1 | 2.96 | 7.03 |
|  |  | (Reference) | (1.18,1.84) | (2.28,3.45) |  | (Reference) | (1.48,2.24) | (2.21,3.42) |  |  |  |  |  | (Reference) | (2.37,3.71) | (5.57,8.92) |
| **Metabolic Syndrome (%)** |  | 12.4 | 19.1 | 33.6 |  | 6.5 | 17.6 | 44.4 |  | 9.7 | 10.6 | 47.9 |  | 5.3 | 17.1 | 46.5 |
| OR |  | 1 | 1.34 | 2.08 |  | 1 | 1.99 | 4.76 |  | 1 | 0.9 | 7.39 |  | 1 | 2.39 | 6.56 |
|  |  | (Reference) | (1.01,1.78) | (1.61,2.69) |  | (Reference) | (1.47,2.73) | (3.54,6.47) |  | (Reference) | (0.65,1.24) | (5.58,9.87) |  | (Reference) | (1.73,3.34) | (4.82,9.05) |
| **2+-non-glycemic and non-anthropometric -criteria (%)** |  | 17.7 | 21 | 31.6 |  | 10.5 | 21.2 | 40.7 |  | 20.2 | 22.1 | 30.2 |  | 10.9 | 21.1 | 40.9 |
| OR |  | 1 | 1.03 | 1.43 |  | 1 | 1.85 | 3.69 |  | 1 | 0.98 | 1.12 |  | 1 | 1.76 | 3.49 |
|  |  | (Reference) | (0.81,1.31) | (1.15,1.78) |  | (Reference) | (1.44,2.39) | (2.87,4.76) |  | (Reference) | (0.78,1.22) | (0.90,1.40) |  | (Reference) | (1.37,2.26) | (2.73,4.48) |
| **Insulin Resistance (%)** |  | 6.5 | 10 | 16.9 |  | **0.0** | **0.0** | **35.1** |  | 2.9 | 8.4 | 23.1 |  | **–** | **–** | **–** |
| OR |  | 1 | 1.41 | 1.95 |  | – | – | –‡ |  | 1 | 2.86 | 7.95 |  | – | – | – |
|  |  | (Reference) | (0.99,2.01) | (1.42,2.71) |  |  |  |  |  | (Reference) | (1.86,4.54) | (5.31,12.35) |  |  |  |  |

Adjusted odds ratios and their confidence interval calculated from a logistic regression model adjusted for age (continuous) and BMI. All tests for linear trend, calculated from models introducing each predictor as continuous variable, were statistically significant at a level α<0.001 except for glucose and high triglycerides (p=0.03) and for glucose and Low HDL-cholesterol (p=0.31). *HOMA-IR: Homeostatic Model Assessment - Insulin Resistance. †OR: Odds Ratio. ‡Outcome variable directly related to the predictor because of a formula or because of a diagnostic criterion.
